# LncRNA ENSMUST00000155383 is Involved in the Improvement of DPP-4 Inhibitor MK-626 on Vascular Endothelial Function by Modulating Cacna1c-Mediated Ca^2+^ Influx in Hypertensive Mice

**DOI:** 10.3389/fmolb.2021.724225

**Published:** 2021-07-23

**Authors:** Yi Zhang, Na Tan, Yi Zong, Li Li, Yan Zhang, Jian Liu, Xiaorui Wang, Wenwen Han, Limei Liu

**Affiliations:** ^1^ Department of Physiology and Pathophysiology, School of Basic Medical Sciences, Peking University, Beijing, China; ^2^ Key Laboratory of Molecular Cardiovascular Science, Ministry of Education, Beijing, China; ^3^ Department of Integration of Chinese and Western Medicine, School of Basic Medical Sciences, Peking University, Beijing, China

**Keywords:** calcium, endothelial function, glucagon-like peptide-1, hypertension, long noncoding RNA

## Abstract

**Objective:** This study investigated the protective effects of dipeptidyl peptidase-4 inhibitor MK-626 on vascular endothelial function by regulating lncRNAs in hypertensive vasculature.

**Methods:** Angiotensin Ⅱ (Ang Ⅱ)-loaded osmotic pumps were implanted in mice with or without MK-626 administration. GLP-1 levels in plasma were measured by ELISA. Aortic rings were suspended in myograph for tension measurement. Microarray was performed to analyze lncRNA and mRNA expression profiles. Protein expression and phosphorylation were examined by Western blot. The differentially expressed (DE)-genes were validated by qRT-PCR. The intracellular Ca^2+^ concentration was detected by laser confocal system.

**Results:** MK-626 elevated plasma GLP-1 level, increased eNOS phosphorylation, improved endothelium-dependent relaxations, and reduced systolic blood pressure in Ang Ⅱ-induced hypertensive mice. Microarray revealed the dysregulations of 723 lncRNAs and 742 mRNAs were reversed by MK-626 in hypertensive mouse aortae. qRT-PCR validation showed that 13 DE-lncRNAs and eight dysregulated mRNAs in both hypertensive mouse aortae and mouse aortic endothelial cells (MAECs) were rescued by MK-626. Among them, four mRNAs (*Cacna1C*, *Itgav*, *Itga8*, and *Npnt*) were co-expressed with lncRNA ENSMUST00000155383. Cacna1C protein expression was reduced in the ECs but was elevated in smooth muscle cells from Ang Ⅱ-infused mice, which were both reversed by MK-626. Knockdown of lncRNA ENSMUST00000155383 suppressed the increased Cacna1c protein and mRNA expression, elevated Ca^2+^ level, and enhanced eNOS phosphorylation induced by MK-626 in the hypertensive mouse ECs.

**Conclusion:** The dysregulations of lncRNA ENSMUST00000155383-associated genes might play crucial roles in hypertension-induced endothelial dysfunction through affecting calcium pathway. MK-626 might ameliorate endothelial dysfunction by upregulating lncRNA ENSMUST00000155383, enhancing Ca^2+^ concentration, and subsequently restoring eNOS activity in hypertension.

## Introduction

Hypertension is one of the most common diseases threatening to human health and is the main risk factor of cardiovascular complications (Li et al., 2017). Endothelial dysfunction is a hallmark of vascular injury in hypertension. The sustained hypertension can damage the functional properties of vascular endothelial cells and result in decreased bioavailability of endothelium-derived nitric oxide (NO), leading to endothelial dysfunction (Konukoglu and Uzun, 2017; Zhao et al., 2019). Meanwhile, endothelial dysfunction is the crucial and initial step in vascular events in hypertension. Thus, the improvement of vascular endothelial function has become a new therapeutic strategy for hypertension-related cardiovascular diseases.

MK-626, a novel dipeptidyl peptidase-4 (DPP-4) inhibitor, is an analog of des-fluoro sitagliptin and plays an important role in controlling glycemia by inhibiting the inactivation and degradation of glucagon-like peptide-1 (GLP-1) and restoring pancreatic β cell secretory function (Liu et al., 2016; Scott, 2017). In recent years, emerging evidence suggests that GLP-1 and related agents exert protective effects in the cardiovascular system (Ban et al., 2008; Kodera et al., 2011). Elevation of GLP-1 by DPP-4 inhibition protects rats against pulmonary vascular and right ventricular remodeling in pulmonary hypertension (Wang et al., 2019). GLP-1 analog liraglutide ameliorates endothelial dysfunction through reducing oxidative stress and prohibiting endothelial nitric oxide synthase uncoupling in angiotensin Ⅱ (Ang Ⅱ)-induced hypertensive mice (Helmstädter et al., 2020). Our previous studies demonstrate that sitagliptin improves endothelial function by restoring NO bioavailability in the renal arteries of spontaneously hypertensive rats (SHRs) (Liu et al., 2012; Liu et al., 2014). However, the molecular mechanisms whereby GLP-1 and its analogues improve vascular endothelial function in hypertension remain largely unknown.

Long non-coding RNAs (lncRNAs), a class of non-coding regulatory RNAs with a length of more than 200 nucleotides, are rapidly emerging as a potential drug therapeutic target (Mercer et al., 2009). LncRNAs regulate gene expression at the transcriptional and post-transcriptional levels and rarely encode proteins (Noh et al., 2018), which play a crucial role in epigenetic control (Muers, 2011) and have arisen as key players in cardiovascular diseases, such as hypertension (Rafiee et al., 2018). One study reveals that lncRNA sONE is markedly increased in renal vascular endothelial cells of high-salt diet-induced hypertensive rats (Zhang et al., 2015). LncRNA AK094457 is significantly upregulated in SHR aortic endothelial cells and aggravates Ang Ⅱ-mediated endothelial dysfunction (Zhuo et al., 2019); while knockdown of lncRNA AK094457 alleviates Ang Ⅱ-induced vascular endothelial cell injury (Xu et al., 2020).

The present study aimed at investigating whether GLP-1 elevation by MK-626 protects against vascular endothelial dysfunction by modulating lncRNA expression profile in the aortae from hypertensive mice, thus providing a therapeutic profile of GLP-1 and its agents against hypertension.

## Methods and Materials

### Animals and Drugs

Male C57BL/6J mice (8–10 weeks old) were purchased from Experimental Animal Center of Peking University Health Science Center. All the experimental protocols in this study were approved by Animal Experimentation Ethics Committee of Peking University Health Science Center and were in accordance with the Guide for the Care and Use of Laboratory Animals of the US National Institutes of Health (NIH Publication, 8th Edition, 2011). All mice were subcutaneously implanted osmotic minipumps for Ang Ⅱ (1 mg/kg/day, Tocris Bioscience, Bristol, United Kingdom) or PBS delivery under ketamine/xylazine anesthesia (75 and 6 mg/kg body weight) and then received MK-626 (Merck, United States) administration (3 mg/kg/day) or vehicle for 2 weeks. Exendin-4 and exendin 9–39 were purchased from Sigma-Aldrich Chemical (St Louis, MO, United States).

### Blood Pressure Measurement

Systolic blood pressure of mice was measured by the tail-cuff method before and after Ang Ⅱ with or without MK-626 administration for 2 weeks. Blood pressure was calculated from the average of three to five successive recordings.

### Measurement of GLP-1 in Plasma

Mice were sacrificed by CO_2_ suffocation and then plasma was collected. GLP-1 levels in plasma were measured by Glucagon-Like Peptide-1 (Active) ELISA kit (Linco Research, Inc, United States) according to the manufacturer’s instruction.

### Aorta Preparation and Functional Assay

Aortae of mice were removed and then placed in ice-cold Krebs solution (mmol/L): 119 NaCl, 25 NaHCO_3_, 4.7 KCl, 1.2 KH_2_PO_4_, 2.5 CaCl_2_, 1 MgCl_2_, and 11 D-glucose. Aortae were cleaned of adhering tissues and cut into ring segments with a length of ∼2 mm. Each aortic ring was suspended in wire myograph (Danish Myo Technology, Aarhus, Denmark) for recording of changes in isometric tension. Each ring was initially contracted by 60 mmol/L KCl. Endothelium-dependent relaxations to acetylcholine (ACh, 0.003–10 μmol/L) were detected in the rings pre-contracted with phenylephrine (Phe, 1 μmol/L). After washing 4 times, the rings were incubated with NG-nitro-L-arginine methyl ester (L-NAME, 100 µmol/L) for 30 min. Then endothelium-independent relaxations to sodium nitroprusside (SNP, 0.001–10 μmol/L) were examined after pre-contracting with Phe. ACh, L-NAME, SNP, and Phe were purchased from Sigma-Aldrich Chemical (St Louis, MO, United States).

### Western Blot Analysis

Isolated aortae, aortic endothelial cells, and smooth muscle cells from mice were homogenized in RIPA lysis buffer (1 μg/ml leupeptin, 5 μg/ml aprotinin, 1 mmol/L sodium orthovanadate, 1 mmol/L sodium fluoride, 100 μg/ml PMSF, 1 mmol/L EGTA, 1 mmol/L EDTA, and 2 μg/ml β-glycerolphosphate) for 30 min, and centrifuged at 12,000 rpm for 20 min at 4°C. Protein lysates (10 μg) were subjected to electrophoresis and transferred to PVDF membrane (Millipore, MA, United States). Blots were blocked with 5% nonfat milk or 1% bovine serum albumin for 1 h and incubated with various primary antibodies overnight at 4°C. The anti-phospho-eNOS (Ser^1177^) and anti-eNOS antibodies were purchased from Cell Signaling Technology (Beverly, MA, United States). Antibody against GAPDH and Cacna1C were from Bioworld Technology (Louis Park, MN, United States). Antibody against β-actin was from Biodragon Technology (Beijing, China). After washing, blots were incubated with HRP-conjugated swine anti-rabbit or anti-mouse IgG. Immunoreactive bands were visualized by ECL reagents (Biodragon, Beijing, China) and imaged with image Lab (Bio-Rad Laboratories, Inc, United States).

### RNA Extraction and LncRNA Microarray

Total RNA was extracted from fresh aortae using TRizol reagent (Invitrogen, CA, United States). The RNA concentration and quality were measured using a Nano Drop ND-1000 (Thermo, MA, United States). The standard denatured agarose gel electrophoresis was used to estimate RNA integrity. Microarray analysis was carried out by KangChen Bio-tech (Shanghai, China). This experiment adapted the Arraystar Mouse LncRNA Microarray Version 3.0. Sample preparation and microarray experiments including labeling and hybridization were carried out according to the manufacturer's protocol.

### Microarray Data Analysis

The acquired array images were analyzed by Agilent Feature Extraction software (version 11.0.1.1). The GeneSpring GX v12.1 software package (Agilent Technologies) was adapted to manipulate quantile normalization and succedent data processing. Differentially expressed (DE)-lncRNAs and mRNAs were selected with a fold change >2 and *p* value < 0.05. Volcano plot filtering was used to identify DE-lncRNAs and DE-mRNAs. Hierarchical Clustering was performed to discriminate the expression patterns of DE-lncRNAs and DE-mRNAs. These sequence data in the present study have been submitted to the NCBI GenBank databases under accession number GSE165561 (https://www.ncbi.nlm.nih.gov/geo/query/acc.cgi?acc=GSE165561)

### Primary Culture of Mouse Aortic Endothelial Cells (MAECs)

Mice were anesthetized with an intraperitoneal injection of ketamine/xylazine. The aortae were placed in DMEM, cleaned of adhering tissues, and then cut along the longitudinal axis. After digestion by 0.2% collagenase type IA for 8 min at 37°C, DMEM (10% FBS) was added and cells were collected by centrifugation at 1,000 rpm for 5 min. Next, the pellet was gently resuspended in DMEM supplemented with 20% FBS and cultured in a 60 mm cell culture dish for 45 min incubation at 37°C. Then, medium was changed by EGM supplemented with bovine brain extract (Lonza, Walkersville, MD) and 10% FBS to remove other cell types. The cells were maintained until 80% confluence.

### Primary Culture of Mouse Aortic Smooth Muscle Cells (SMCs)

After removed adhering tissue, the isolated aortae were cut along the longitudinal axis. The vascular intima was carefully wiped with ophthalmic forceps to scrape off vascular endothelium. Next, the aortae were cut into tissue blocks of approximately 1 mm × 1 mm, evenly planted on the bottom of the 35 mm cell culture dish, and then placed in the cell incubator with 5% CO_2_ at 37°C. After the tissue blocks were firmly attached to the bottom of the dish, 2 ml DMEM containing 20% FBS was slowly added and the tissue blocks were placed in the incubator. The medium was changed every 3 days and the cells were maintained until 80% confluence before use.

### Quantitative Real-Time PCR Validation

RNA from aortae or endothelial cells was reversely transcribed into cDNA using NovoScript® 1st Strand cDNA Synthesis Super Mix (Novoprotein, Shanghai, China). Quantitative real time polymerase chain reaction (qRT-PCR) was carried out by Thermo Fisher Piko real-time PCR system using SYBR® qPCR Super Mix (NovoStart®, Shanghai, China). All experiments were conducted at least 3 times. The 2^-ΔΔCt^ method was used to evaluate the lncRNA or mRNA expressions and the calculation was normalized to GAPDH expression. All primers were listed in supplementary Table 1 (lncRNAs) and supplementary Table 2 (mRNAs).

### GO and KEGG Pathway Analyses

Gene ontology (GO) (http://www.geneontology.org) is a comprehensive resource in molecular and cellular biology for cataloguing gene function, which covers three aspects including cellular components (CC), molecular functions (MF), and biological processes (BP). Kyoto Encyclopedia of Genes and Genomes (KEGG) (http://www.genome.jp/kegg/) analysis is a database resource for interpreting the functional meanings to genes and genomes and analyzing the related pathways. GO enrichment and KEGG pathway analyses, therefore, were performed to identify the functions of differentially expressed genes in this study.

### Coding-Noncoding Co-Expression Analysis

The coding-noncoding gene co-expression network (CNC) was constructed to identify the interactions between differentially expressed lncRNAs and their co-expressed mRNAs. A hybrid hierarchical clustering algorithm was performed to analyze the relationship between lncRNA and mRNA and to calculate the Pearson correlation coefficient (PCC) of each pair. The lncRNA-mRNA pairs with a PCC >0.90, *p* value ≤ 0.05, and false discovery rate (FDR) ≤ 1 were identified and chosen to construct a network. The network was drawn using Cytoscape software (Version 3.6.0). GO and KEGG analyses were further performed for targeted-mRNAs to characterize the CNC network fully.

### Transfection Condition

Small interfering RNA (siRNA) targeting lncRNA ENSMUST00000155383 and negative control (NC) were obtained from HanBio (Shanghai, China). Endothelial cells were seeded in 6-well plates at 60–70% confluence and transfected with ENSMUST00000155383 siRNA (100 nmol/L) or corresponding NC by using Lipofectamine RNAiMax reagent (Invitrogen, CA, United States). Cells were collected for various measurements 48 h after transfection.

### Measurement of Cytosolic Ca^2+^ Concentration

MAECs seeded on the confocal dish were loaded with 5 μmol/L Ca^2+^ fluorescence probe Fluo-4/AM (Invitrogen, CA, United States) for 40 min at 37°C in normal physiological saline solution (NPSS, mmol/L): 140 NaCl, 1 CaCl_2_, 5 HEPES, 5 KCl, 1 MgCl_2_·6H_2_O, and 10 D-Glucose. Cells were then washed 3 times with NPSS and incubated for further 20 min at room temperature. Fluorescence images were captured by using a confocal microscope (Leica, German) with excitation at 488 nm and emission at 505–530 nm. Data from 80 individual cells were collected each group.

### Statistical Analysis

All data were analyzed with IBM SPSS Statistics 24.0 software. Differential expressions of lncRNAs and mRNAs were compared by one-way ANOVA within the three groups. GO and KEGG pathway analysis were evaluated using Fisher’s exact test. All values were presented as the mean ± SEM, and significance was assigned at the *p* < 0.05.

## Results

### MK-626 Reduces Blood Pressure and Ameliorates Endothelial Dysfunction in Ang Ⅱ-Induced Hypertensive Mice

**FIGURE 1:**
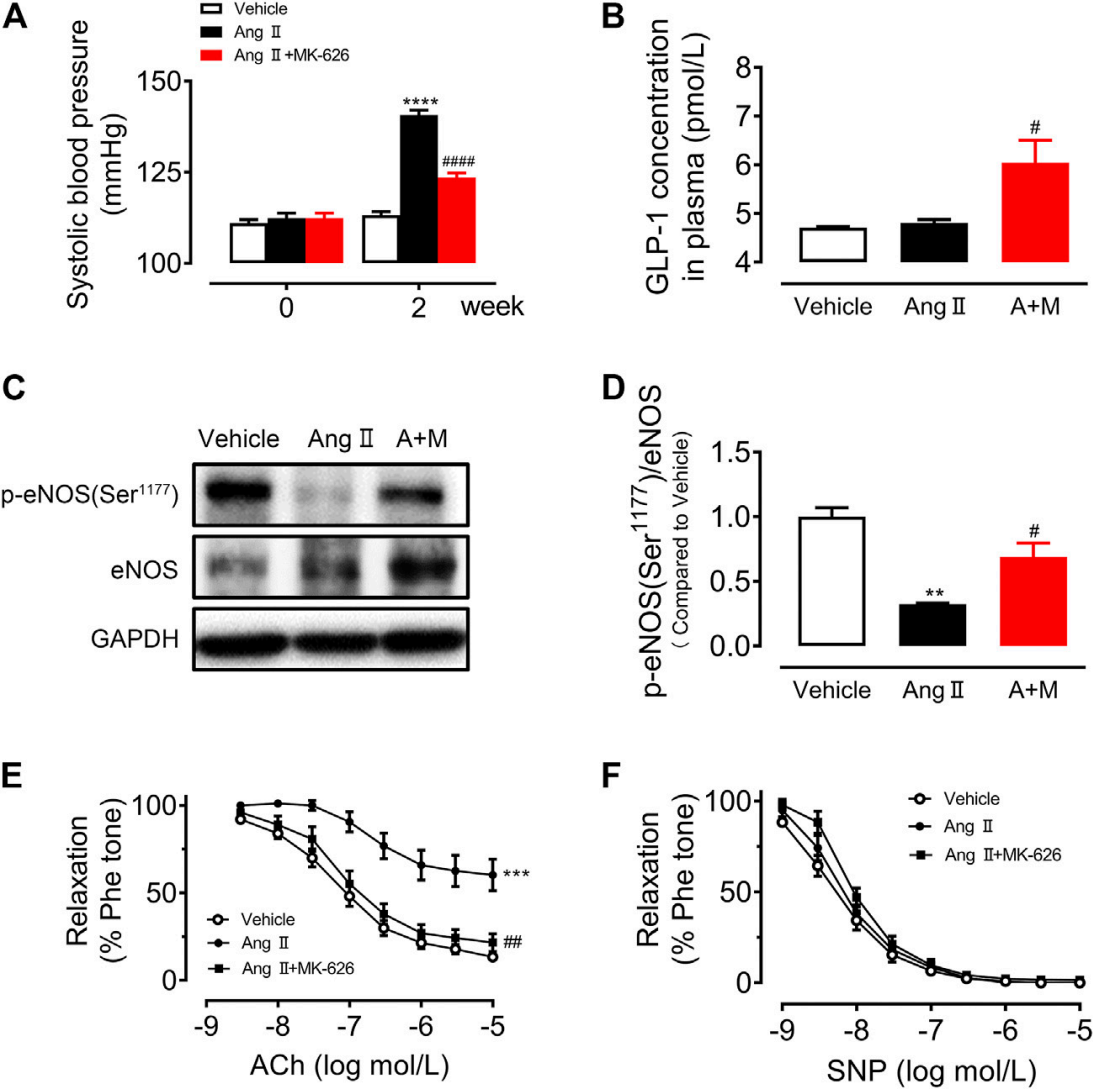
MK-626 decreases systolic blood pressure and improves aortic endothelial dysfunction in Ang Ⅱ-induced hypertensive mice. **(A)** Systolic blood pressure in Vehicle, Ang Ⅱ and Ang Ⅱ + MK-626 co-treatment mice. **(B)** GLP-1 levels in mouse plasma. **(C–D)** Levels of eNOS phosphorylation and expression in the mouse aortae. ACh-induced endothelium-dependent relaxations (**E**) and SNP-induced endothelium-independent relaxations **(F)** in the aortae from mice. ^**^ *p* < 0.01, ^***^ *p* < 0.001, ^****^ *p* < 0.0001 *vs* Vehicle; ^#^ *p* < 0.05, ^##^ *p* < 0.01, ^####^ *p* < 0.0001 *vs* Ang Ⅱ. Data are expressed as mean ± SEM (*n* = 4 for Western blot, *n* = 5–7 for other experiments). A + M, Ang Ⅱ + MK-626.

To verify the anti-hypertensive effect of DPP-4 inhibitor MK-626 in hypertension, we first measured the systolic blood pressure (sBP) before and after 2 weeks Ang Ⅱ infusion with or without MK-626 treatment. As shown in Figure 1A, Ang Ⅱ induced a significant elevation of sBP compared with Vehicle, indicating a successful establishment of hypertensive animal model in mice. As the meanwhile, MK-626 co-treatment lowered sBP in hypertensive mice. Since DPP-4 inhibitors exert biological effects through elevating GLP-1, we examined GLP-1 levels in plasma by ELISA. The result showed that there was no difference between Vehicle and Ang Ⅱ-infused mice. However, MK-626 significantly increased plasma GLP-1 level in hypertensive mice (Figure 1B). In hypertension, reduced NO bioavailability correlates with impaired vascular function. Thus, we examined the level of endothelial nitric oxide synthesis (eNOS) phosphorylation. We revealed that Ang Ⅱ resulted in the reduced eNOS phosphorylation (Figures 1C,D) and the impaired endothelium-dependent relaxations (Figure 1E) in mouse aortae, but MK-626 alleviated the injuries caused by Ang Ⅱ (Figures 1C–E). In addition, endothelium-independent relaxations to sodium nitroprusside (SNP) were of no significance in three groups (Figure 1F). These data suggest that MK-626 reduces blood pressure most likely through ameliorating vascular endothelial dysfunction in Ang Ⅱ-induced hypertensive mice.

### MK-626 Regulates the Expression Profiles of lncRNAs and mRNAs in Hypertensive Mouse Aortae

**FIGURE 2:**
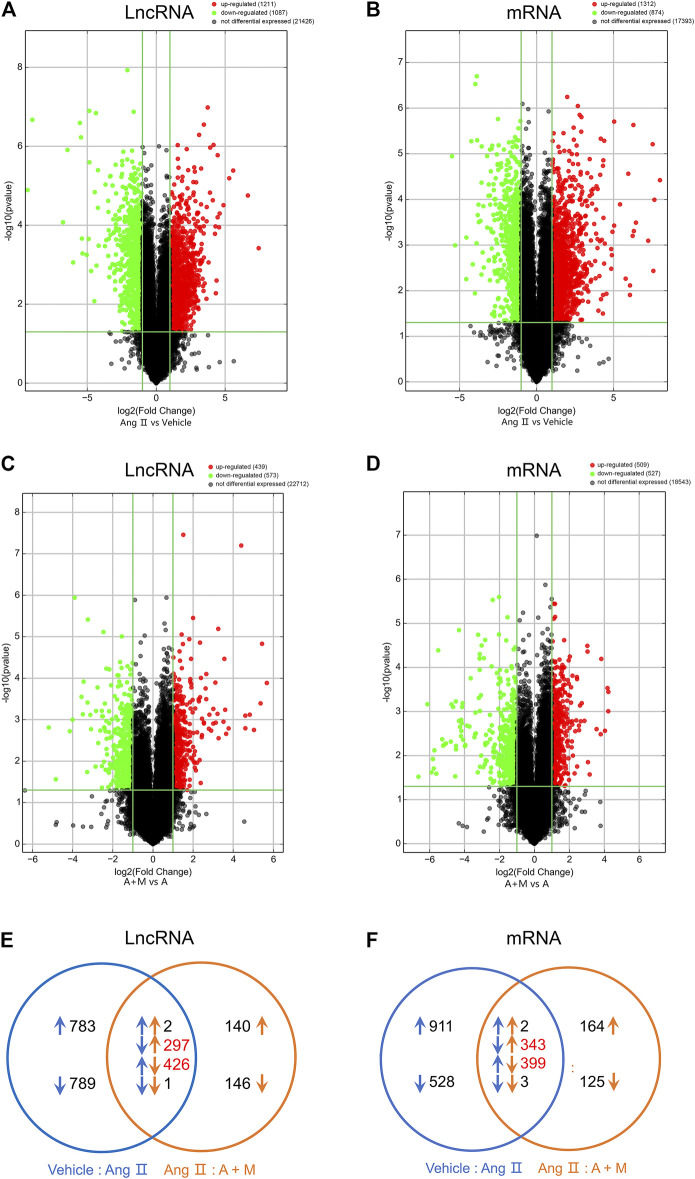
MK-626 regulates expression profiles of lncRNAs and mRNAs. Volcano plot for differentially expressed lncRNAs and mRNAs in Ang Ⅱ group compared with Vehicle **(A–B)** and A + M group compared with Ang Ⅱ group **(C–D)**. The red points represent upregulated genes; the green points represent downregulated genes; the black points represent unchanged genes. **(E–F)** A Venn diagram of differentially expressed lncRNAs and mRNAs in the mice. The comparison of A + M group and Ang Ⅱ group (orange arrows); the comparison of Ang Ⅱ group and Vehicle group (blue arrows); the upregulated genes (upward arrows); the downregulated genes (downward arrows); the number of genes regulated by MK-626 (red number). A + M, Ang Ⅱ + MK-626.

So far, a few lncRNAs have been implicated in modulating vascular endothelial function. To investigate the functional lncRNAs associated with hypertension and clarify the therapeutic mechanism of MK-626, the microarray was used to analyze expression profiles of the lncRNAs and mRNAs in the aortae from the three groups. The microarray analysis identified that 2,298 lncRNAs (Figure 2A) and 2,186 mRNAs (Figure 2B) were differentially expressed in Ang Ⅱ group compared with Vehicle group, however, 1,012 lncRNAs (Figure 2C) and 1,036 mRNAs (Figure 2D) were obviously changed in Ang Ⅱ plus MK-626 treatment group compared with Ang group. As shown in volcano plots (Figures 2A,B), 1,211 lncRNAs and 1,312 mRNAs were upregulated (fold change >2.0 and *p* value <0.05) but 1,087 lncRNAs and 874 mRNAs were significantly downregulated in Ang Ⅱ group compared with Vehicle group. Meanwhile, 573 lncRNAs and 527 mRNAs were downregulated whilst 439 lncRNAs and 509 mRNAs were markedly upregulated in Ang Ⅱ and MK-626 co-treatment group compared with Ang Ⅱ group (Figures 2C,D).

To figure out the extent to which MK-626 regulated gene expressions in hypertension, we conducted Venn analysis on the overlapped and DE-lncRNAs and DE-mRNAs. Among the downregulated lncRNAs induced by Ang Ⅱ, 297 lncRNAs were rescued by MK-626; while 426 lncRNAs upregulated by Ang Ⅱ were suppressed by MK-626 (Figure 2E). In the meantime, MK-626 significantly downregulated 399 mRNAs enhanced by Ang Ⅱ and upregulated 343 mRNAs diminished by Ang Ⅱ (Figure 2F). Subsequently, hierarchical clustering analysis showed that 60 most significantly DE-lncRNAs and DE-mRNAs (30 upregulated and 30 downregulated) were reversed by MK-626 in hypertensive mice (Supplementary Figure S1). Taken together, the present results indicate that MK-626 might improve endothelial function by regulating expression profiles of lncRNAs and mRNAs in Ang Ⅱ-induced hypertension.

### Validation and Functional Analysis of the Dysregulated lncRNAs Possibly Involved in the Regulatory Actions of MK-626 on Vascular Endothelial Function in Hypertension

**FIGURE 3:**
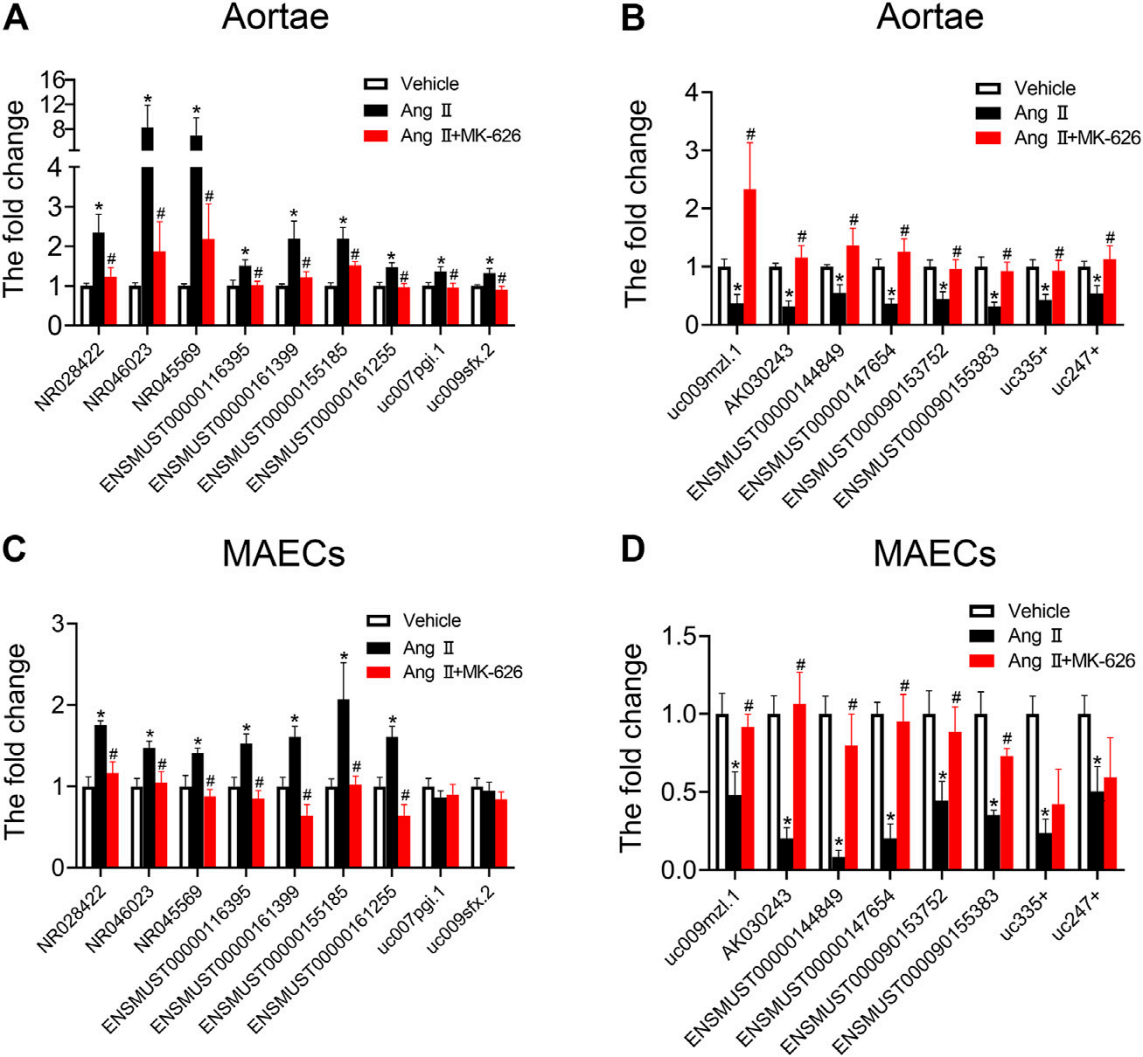
Quantitative real-time PCR validation. qRT-PCR validated the expression of upregulated and downregulated lncRNAs in the mouse aortae **(A–B)** and aortic endothelial cells **(C–D)**. ^*^ *p* < 0.05 *vs* Vehicle; ^#^ *p* < 0.05 *vs* Ang Ⅱ. Data are expressed as mean ± SEM (*n* = 6–9 for aortae, *n* = 4–6 for endothelial cells).

To validate the accuracy of microarray analysis and expound the possible regulatory actions of dysregulated lncRNAs on vascular function, according to the fold change, we selected 24 dysregulated lncRNAs (14 upregulated lncRNAs and 10 downregulated lncRNAs) among the lncRNAs altered by Ang Ⅱ but modulated by MK-626 for qRT-PCR validation. Of these, nine of 14 upregulated lncRNAs (Figure 3A) and eight of 10 downregulated lncRNAs (Figure 3B) by Ang Ⅱ were confirmed to be reversed by MK-626, consistent with the microarray data. In order to further confirm whether the 17 lncRNAs were involved in the improvement of MK-626 on hypertension-induced endothelial dysfunction, we verified the levels of these lncRNAs in cultured primary mouse aortic endothelial cells (MAECs) from the three groups by qRT-PCR. As shown in Figures 3C,D, 13 of 17 lncRNAs exhibited similar changes to those from aortae except uc007pgi.1, uc009sfx.2, uc335+, and uc247+.

To delve deeper into the molecular basis of MK-626 in regulating lncRNAs to ameliorate endothelial dysfunction induced by Ang Ⅱ, we acquired co-expressed coding genes for 13 lncRNAs mentioned above by calculating Person coefficient with PCC >0.90, *p* value ≤ 0.05 and FDR ≤ 1. Through GO analysis, 1853 biological process (BP) terms, 134 cellular component (CC) terms, and 152 molecular function (MF) terms were found in DE-genes. The top 10 terms of BP, CC and MF were listed in Supplementary Figure S2. Our results indicated that the meaningful BP terms were associated with regulation of multicellular organismal process, cell communication, and regulation of localization. The DE-lncRNAs of CC were obviously enriched in cell periphery and plasma membrane. Whereas the terms in MF suggested that protein binding and binding seemed to be particularly important in the improvement of MK-626 on vascular endothelial function.

### Construction of LncRNA-mRNA Co-Expression Network Regulated by MK-626

**FIGURE 4:**
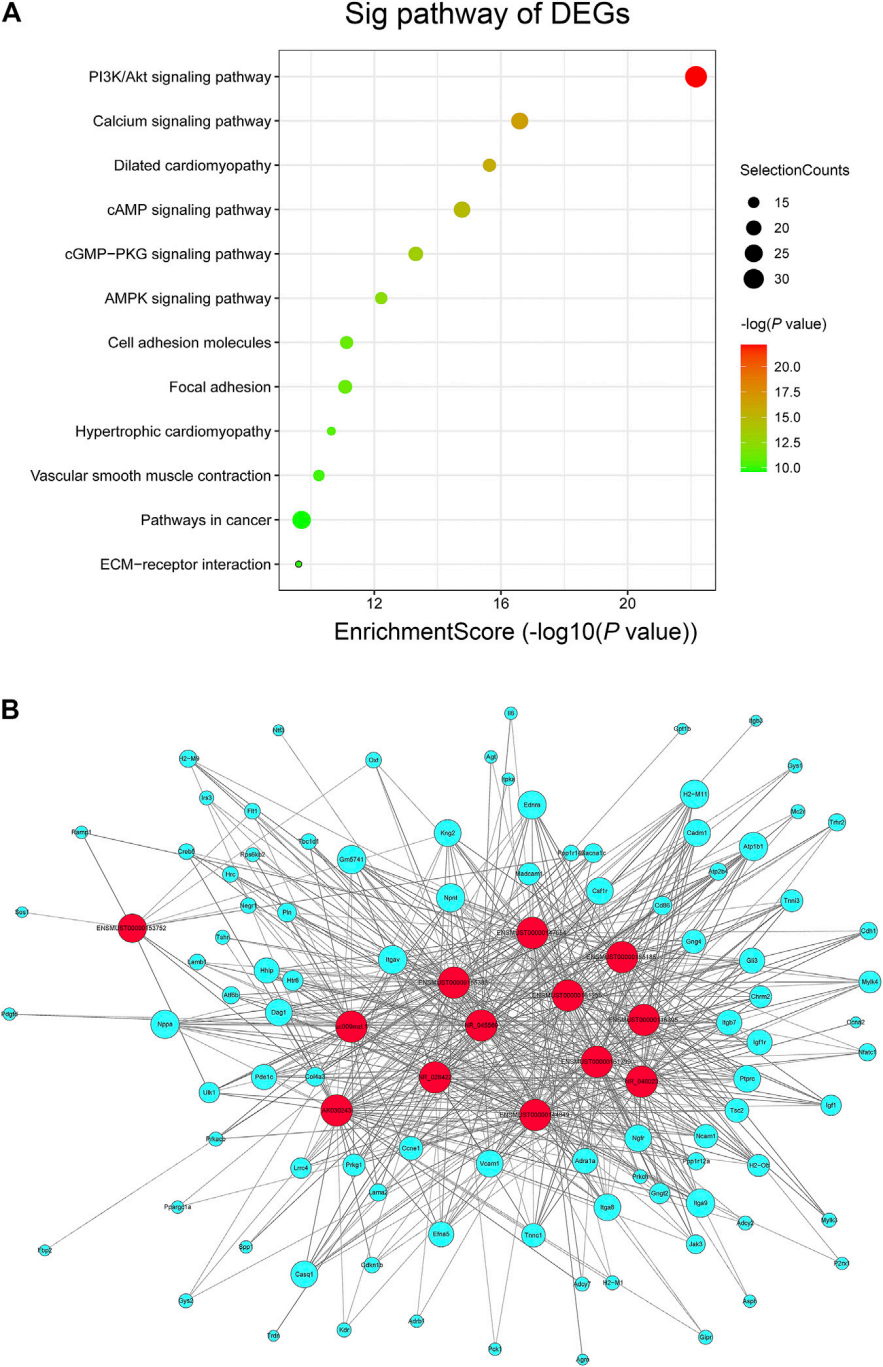
LncRNA-mRNA co-expression network. **(A)** KEGG pathway enrichment analysis of differentially expressed genes (DEGs) involved in the vascular regulation. **(B)** lncRNA-mRNA network to investigate the beneficial effects of MK-626 on hypertension-induced endothelial function. The red and lake blue nodes correspond to lncRNAs and coding genes respectively. The solid lines represent positive correlation and dotted lines represent negative correlation.

In order to reveal the potential signaling pathways participated in the regulation of MK-626 on endothelial function in Ang Ⅱ-induced hypertensive mice, we performed KEGG analysis. Based on the Enrichment Score = −log10 (*p* value) and *p* value < 0.05, the co-expressed genes were significantly enriched in 112 pathways. The top 12 enriched pathways related to co-expressed coding genes were showed in Figure 4A. Among the top 12 pathways, a total of nine pathways including PI3K/Akt signaling pathway, calcium signaling pathway, cAMP signaling pathway, cGMP-PKG signaling pathway, AMPK signaling pathway, cell adhesion molecules, focal adhesion, vascular smooth muscle contraction, and ECM-receptor interaction signaling pathway were previously reported to be associated with the regulation of vascular function.

To further explore the molecular mechanism of MK-626 in the regulation of lncRNAs to reverse endothelial dysfunction in hypertension, we recalculated the co-expressed genes for 13 DE-lncRNAs in accordance with the nine signaling pathways. The lncRNA-mRNA co-expression network was therefrom constructed to accurately and deeply investigate the beneficial effects of MK-626 on endothelial function in hypertension. 571 pairs of lncRNA-mRNA co-expression relationships were found according to the selected 13 lncRNAs and 95 mRNAs (Figure 4B). Importantly, lncRNA ENSMUST00000155383 was co-expressed with the maximum numbers of 69 genes in the coding non-coding co-expression (CNC) network (Supplementary Table 3), suggesting that this lncRNA might play an important role in regulating vascular endothelial function in hypertension.

### Identification of mRNAs and Signaling Pathways in LncRNA-mRNA Co-Expression Network

**FIGURE 5:**
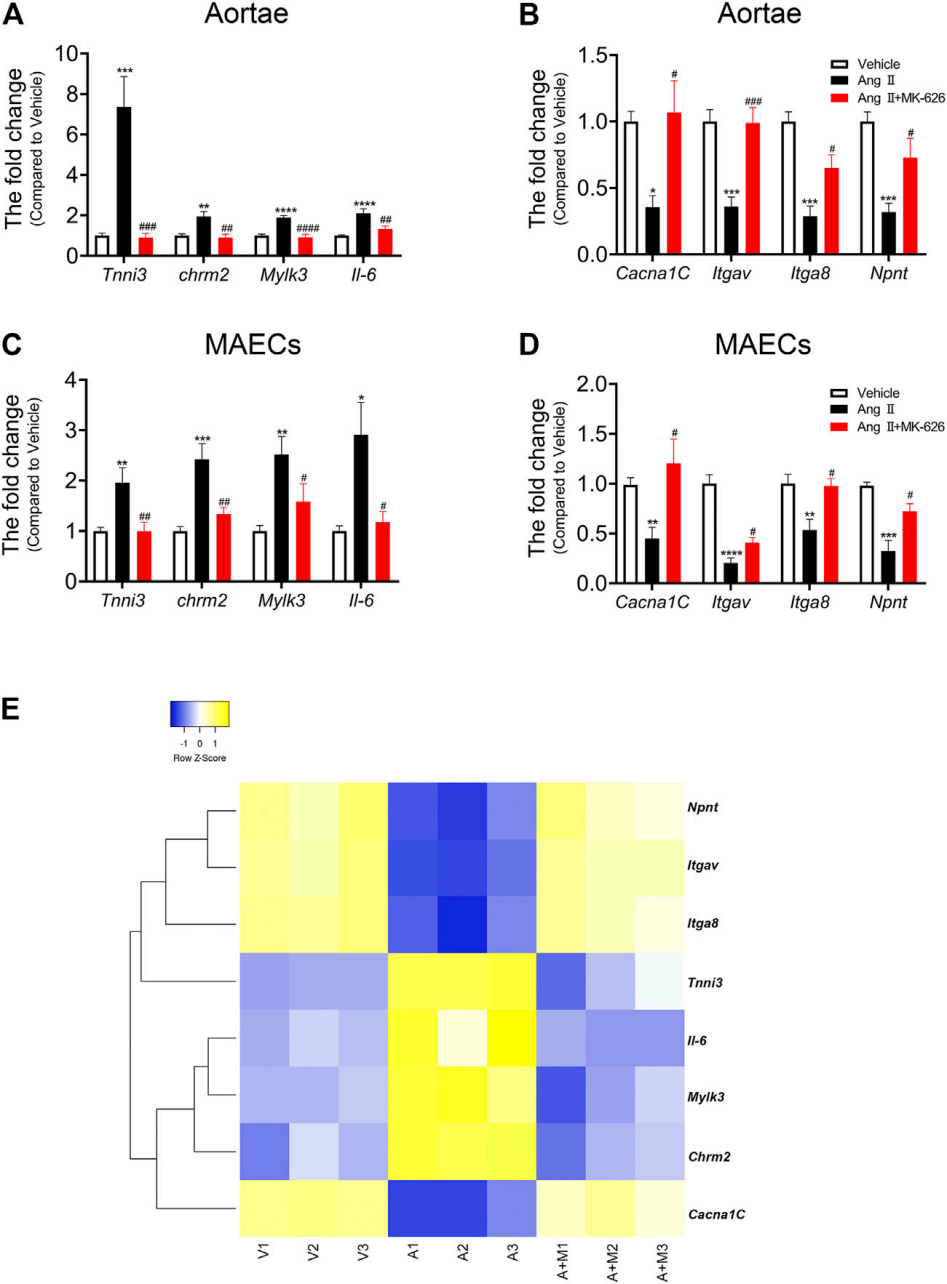
mRNA verification and heatmap analysis. qRT-PCR validated the expression of eight candidate mRNAs in the aortae **(A–B)** and MAECs **(C–D)**. **(E)** Heatmap for eight candidate mRNAs. ^*^ *p* < 0.05, ^**^ *p* < 0.01, ^***^ *p* < 0.001, ^****^ *p* < 0.0001 *vs* Vehicle; ^#^ *p* < 0.05, ^##^ *p* < 0.01, ^###^ *p* < 0.001, ^####^ *p* < 0.0001 *vs* Ang Ⅱ. Data are expressed as mean ± SEM (*n* = 6–9 for aortae, *n* = 4–6 for endothelial cells). V, Vehicle; A, Ang Ⅱ; A + M, Ang Ⅱ + MK-626; MAECs, mouse aortic endothelial cells.

In the light of both our constructed lncRNA-mRNA co-expression network and already-published research related to vascular function, we selected six mRNAs (*Tnnc1*, *Trdn*, *Tnni3*, *Chrm2*, *Mylk3*, and *Il-6*) upregulated and 5 mRNAs (*Ulk1*, *Cacna1C*, *Itgav*, *Itga8*, and *Npnt*) downregulated by Ang Ⅱ but modulated by MK-626 for qRT-PCR validation. Among the six upregulated mRNAs by Ang Ⅱ, the levels of *Tnni3*, *Chrm2*, *Mylk3*, and *Il-6* were suppressed by MK-626 in mouse aortae (Figure 5A). Four of 5 downregulated mRNAs by Ang Ⅱ in the aortae of mice, *Cacna1C*, *Itgav*, *Itga8*, and *Npnt*, were rescued by MK-626 treatment (Figure 5B). These results were consistent with microarray data. In order to further confirm the key roles of these eight targeted mRNAs in the improvement of MK-626 on endothelial function in hypertension, we isolated and cultured primary MAECs of the three groups. qRT-PCR validation exhibited the similar changes to those from aortae (Figures 5C,D). In addition, clustering heatmap analysis revealed the eight mRNA expression profiles among the three groups (Figure 5E and Supplementary Table 4), suggesting that MK-626 probably ameliorated aortic endothelial dysfunction by suppressing the expressions of *Tnni3*, *Chrm2*, *Mylk3*, and *Il-6* and restoring the expressions of *Cacna1C*, *Itgav*, *Itga8*, and *Npnt* in Ang Ⅱ-induced hypertensive mice.

The above results showed that the co-expression pairs of lncRNA ENSMUST00000155383 were predominant in the CNC network. More importantly, lncRNA ENSMUST00000155383 was included in the 13 DE-lncRNAs we identified and it was co-expressed with *Cacna1C*, *Itgav*, *Itga8*, and *Npnt* (seen in Table 1). The pathway-based network analysis of lncRNA ENSMUST00000155383 and the four mRNAs was presented in Supplementary Figure S3. Therefore, we speculated that lncRNA ENSMUST00000155383 was expected to be a regulatory target for the improvement of endothelial function by MK-626 in the vasculature under hypertension.

**TABLE 1:** The co-expression analysis between lncRNA ENSMUST0000055383 and four mRNAs.

LncRNA	mRNA	PCC	PCC type	*p* Value
ENSMUST00000155383	*Cacna1c*	0.964015	+	< 0.0001
	*Itga8*	0.997248	+	< 0.0001
	*Itgav*	0.995858	+	< 0.0001
	*Npnt*	0.995685	+	< 0.0001

PCC, Pearson correlation coefficient, mRNA symbols are italicized.

### LncRNA ENSMUST00000155383 Regulates Endothelial Function Through Affecting Cytosolic Ca^2+^ Concentration and eNOS Activity

**FIGURE 6:**
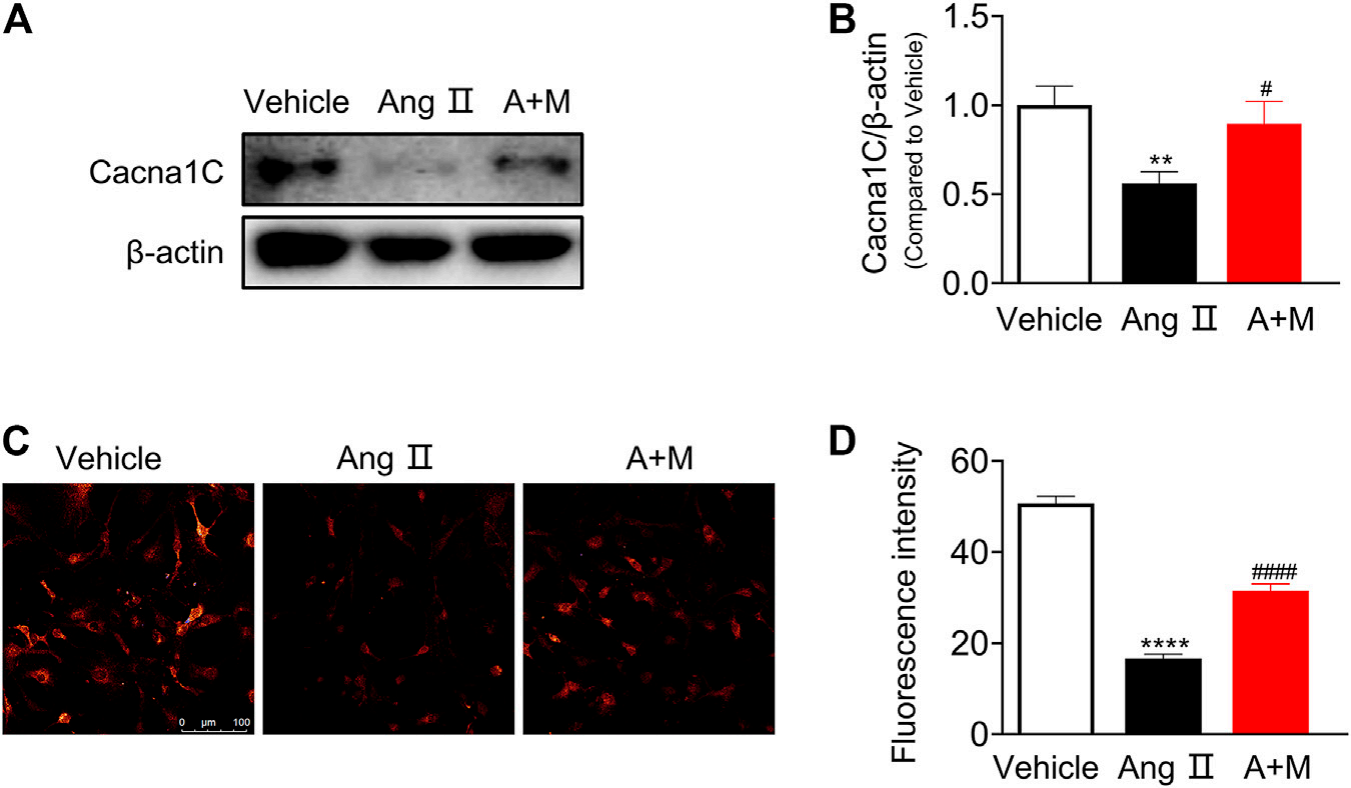
Cacna1c expression and Ca^2+^ level. Protein expressions of Cacna1c were downregulated in the aortic endothelial cells from hypertensive mice, which were rescued by MK-626 **(A–B)**. The reduced Ca^2+^ level was also restored after MK-626 administration in the endothelial cells of hypertensive mice **(C–D)**. ^**^ *p* < 0.01, ^****^ *p* < 0.0001 *vs* Vehicle; ^#^ *p* < 0.05, ^####^ *p* < 0.0001 *vs* Ang Ⅱ. Data are expressed as mean ± SEM (*n* = 4–6 for Western blot; n = 80 individual cells for the measurement of Ca^2+^ level, scale bar = 100 μm). A + M, Ang Ⅱ + MK-626.

Since this study shed light on the critical role of lncRNA ENSMUST00000155383, we preliminarily explored the protective mechanism of this lncRNA on endothelial function in hypertension. Based on the pathway-based network analysis, we found that *Cacna1c* was involved in several vascular function-related pathways including calcium signaling pathway, vascular smooth muscle contraction, cGMP-PKG signaling pathway, and cAMP signaling pathway. First, we examined Cacna1c expressions in the mouse aortic smooth muscle cells (SMCs, Supplementary Figure S4), and endothelial cells (ECs, Figures 6A,B). Western blot demonstrated that the protein levels of Cacna1c was elevated in SMCs after Ang Ⅱ infusion but suppressed by MK-626. However, Ang Ⅱ resulted in the reduction of Cacna1c expression and MK-626 reversed such harmful effect in the ECs (Figures 6A,B). *Cacna1c* gene encodes L-type Ca^2+^ channel Ca_V_1.2 which plays an important role in transient activation of calcium influx. So next, we detected cytosolic Ca^2+^ level in endothelial cells. The intracellular Ca^2+^ levels in the ECs from hypertensive mice were significantly lower than those from Vehicle, while MK-626 administration restored partially the decreased Ca^2+^ concentration (Figures 6C,D).

**FIGURE 7:**
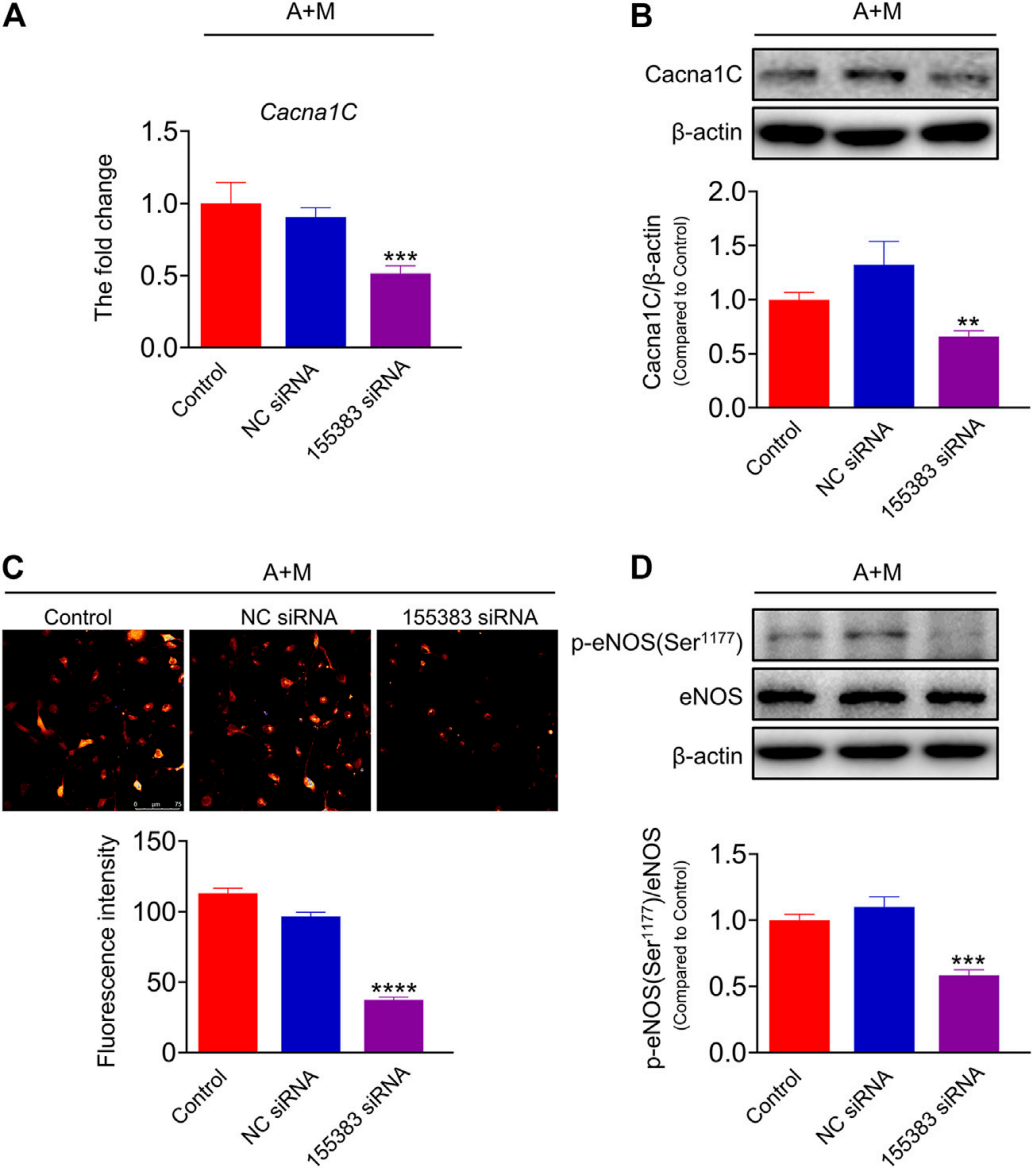
The effects of lncRNA ENSMUST00000155383 silence. Small interfering RNA (siRNA) of lncRNA ENSMUST00000155383 reduced Cacna1c mRNA level **(A)** and protein expression **(B)** in the aortic endothelial cells from hypertensive mice. Silencing lncRNA ENSMUST00000155383 resulted in the reduction of Ca^2+^ level **(C)** and eNOS phosphorylation (**D**) in the MAECs in hypertension. ^**^ *p* < 0.01, ^***^ *p* < 0.001, ^****^ *p* < 0.0001 *vs* NC siRNA. Data are expressed as mean ± SEM. (*n* = 4–6 for Western blot; *n* = 80 individual cells for the measurement of Ca^2+^ level, scale bar = 75 μm). 155383, lncRNA ENSMUST00000155383; A + M, Ang Ⅱ + MK-626.

In order to further clarify the role of lncRNA ENSMUST00000155383 and the regulation of MK-626 on lncRNA ENSMUST00000155383 in hypertension-related endothelial dysfunction, we used siRNA delivery and achieved >60% reduction in the expression of lncRNA ENSMUST00000155383 in the aortic ECs from Ang Ⅱ and MK-626 co-treatment mice (Supplementary Figure S5). Silence of lncRNA ENSMUST00000155383 led to the downregulation of Cacna1c mRNA level (Figure 7A) and protein expression (Figure 7B). Furthermore, the siRNA also caused the decrease of Ca^2+^ concentration in the ECs from hypertensive mice with MK-626 treatment (Figure 7C). Ca^2+^-dependent activation of calmodulin is known to activate eNOS. Thus, we finally investigated the effect of siRNA delivery on eNOS activity in the ECs from hypertensive mice after MK-626 therapy. The result proclaimed that lncRNA ENSMUST00000155383 silencing exactly brought into the reduced eNOS phosphorylation (Figure 7D). These results indicate that lncRNA ENSMUST00000155383 probably restored eNOS activity through rising Cacna1c and subsequently increasing Ca^2+^ entry in the amelioration of MK-626 on endothelial dysfunction.

## Discussion

Hypertension is associated with impaired vascular endothelial function. A number of studies have indicated the protective effects of DPP-4 inhibitors in cardiovascular diseases including hypertension (Diebold et al., 2018; Siraj et al., 2020). The present study suggested that a novel DPP-4 inhibitor MK-626 could ameliorate aortic endothelial dysfunction and lower blood pressure in Ang Ⅱ-induced hypertensive mice through raising GLP-1 level, which was consistent with our previous finding in the renal arteries of SHRs (Liu et al., 2012).

Long noncoding RNAs (lncRNAs), one of the emerging regulators, are involved in multiple biological processes. Recently, the analysis based on expression profiles of lncRNAs and mRNAs has been applied widely to reveal the underlying molecular mechanisms in the pathogenesis of cardiovascular diseases (Cao et al., 2018; Wu et al., 2019). Therefore, we performed a microarray analysis of lncRNAs and mRNAs of the mouse aortae to explore the molecular basis underlying mitigating endothelial dysfunction of MK-626 in hypertension. We here announced that 2,298 lncRNAs and 2,186 mRNAs were differentially expressed in Ang Ⅱ group compared with Vehicle group, whereas, 1,012 lncRNAs and 1,036 mRNAs were differentially expressed after MK-626 co-treatment. Meanwhile, MK-626 modulated 723 lncRNAs and 742 mRNAs which were dysregulated in hypertensive mice, suggesting that MK-626 played a profound action in regulating hypertension-related genes. Among the 723 differentially expressed (DE) lncRNAs regulated by MK-626, 24 candidate lncRNAs were selected for qRT-PCR validation according to fold change, *p* value, and existing research. Subsequent results from the aortae and endothelial cells both showed that seven up-regulated lncRNAs and six down-regulated lncRNAs by Ang Ⅱ were modulated by MK-626, which were consistent with the microarray data. These findings for the first time provide a genetic basis for understanding the pharmaceutical mechanism of GLP-1 in preventing against vascular dysfunction in hypertension. GO and KEGG pathway analyses revealed the potential functions of the DE-genes (DEGs), which were related to the 13 lncRNAs, involved in the therapeutic effects of MK-626. GO analysis showed that the DEGs were significantly enriched in regulation of multicellular organismal process, cell communication, regulation of localization, cell periphery, plasma membrane, protein binding, and binding. KEGG pathway analysis revealed that the DEGs were mainly enriched in 12 signaling pathways, among them, nine pathways related to vascular function were identified, including PI3K/Akt signaling pathway, calcium signaling pathway, cAMP signaling pathway, cGMP-PKG signaling pathway, AMPK signaling pathway, cell adhesion molecules, focal adhesion, vascular smooth muscle contraction, and ECM-receptor interaction.

LncRNAs have long been investigated due to their key roles in modulating a variety of biological or pathological functions through targeting their mRNAs at transcriptional or post-transcriptional level (Huang, 2018). In accordance with the constructed lncRNA-mRNA co-expression network and existing data, we preliminarily selected 11 mRNAs (6 upregulated and 5 downregulated mRNAs induced by Ang Ⅱ but reversed by MK-626) for qRT-PCR validation. The four upregulated mRNAs response to Ang Ⅱ, *Tnni3*, *Chrm2*, *Mylk3*, and *Il-6*, were suppressed by MK-626 both in mouse aortic rings and in cultured primary endothelial cells. The four mRNAs (*Cacna1C*, *Itgav*, *Itga8*, and *Npnt*) downregulated by Ang Ⅱ, meanwhile, were rescued by MK-626 treatment in the aortae and aortic endothelial cells from mice. This part of the results suggested that MK-626 might relieve hypertension-induced endothelial dysfunction by suppressing the expressions of *Tnni3*, *Chrm2*, *Mylk3*, and *Il-6* and restoring the expressions of *Cacna1C*, *Itgav*, *Itga8*, and *Npnt*. Importantly, lncRNA ENSMUST00000155383, which was included in the 13 DE-lncRNAs we have identified, was co-expressed with *Cacna1C*, *Itgav*, *Itga8*, and *Npnt*. Also, our previous study revealed that lncRNA ENSMUST00000155383 mainly expressed in endothelial cells of the vasculature, which was downregulated in hypertensive mouse aortae (Tan et al., 2021). Altogether, we speculated the dysregulation of lncRNA ENSMUST00000155383 and the four related-mRNAs might play an important role in vascular endothelial dysfunction under hypertension. According to the pathway-based network analysis of lncRNA ENSMUST00000155383 and the four mRNAs, we found that *Itgav*, *Itga8*, and *Npnt* participated in the ECM-receptor interaction. *Itgav* and *Itga8* were also related to the PI3K/Akt signaling pathway. The most important reason behind exploring the role of *Cacna1c* in current study is that it was involved in four vascular function-related pathways including calcium signaling pathway, vascular smooth muscle contraction, cGMP-PKG signaling pathway, and cAMP signaling pathway. *Cacna1c* gene encodes L-type Ca^2+^ channel Ca_V_1.2 which can be functionally modulated by vasoactive agents *via* PKC and PKA (Keef et al., 2001). Elevated cGMP levels promote the relaxation of vascular smooth muscle by decreasing Ca^2+^ influx in part through voltage-dependent L-type Ca^2+^ channel (Simard and Li, 2000). Ang Ⅱ upregulates vascular Ca_V_1.2 protein in vascular smooth muscle by stimulating endothelial NAD(P)H oxidase-derived hydrogen peroxide (Wang et al., 2011). We also found that Ang Ⅱ induced the elevation of Cacna1c protein expressions in aortic smooth muscle cells (SMCs) from hypertensive mice. One study has shown that nitric oxide (NO) synthesized by eNOS downregulates the L-type Ca^2+^ channel in the SMCs of basilar artery (Simard and Li, 2000). Furthermore, Ang Ⅱ induced-endothelial dysfunction impaired the regulatory control of L-type Ca^2+^ channels by eNOS (Gerzanich et al., 2003). In this study, the hypertensive mice induced by Ang Ⅱ exhibited the reduced eNOS phosphorylation in the aortae, suggesting that the enhancement of Cacna1c in SMCs of hypertensive mouse aortae was probably due to the impairment of eNOS. The activity of eNOS is regulated by many factors, in which classical activation pathway depends on an increase of intracellular Ca^2+^ concentration and the binding of Ca^2+^/calmodulin (CaM) to the enzyme (Daiber et al., 2019). Acetylcholine stimulated-endothelial NO release can be abolished by chelating extracellular Ca^2+^ or CaM antagonist (Fleming and Busse, 2003; Vanhoutte et al., 2009). Here, we revealed that Cacna1c expression was downregulated and intracellular Ca^2+^ was lowered in the aortic ECs from hypertensive mice, indicating that Ang Ⅱ possibly inhibited eNOS activity partly via suppressing Ca_V_1.2-mediated Ca^2+^ entry in vascular endothelial cells. In addition, our study expected to improve the understanding of the novel therapeutic molecular mechanism of GLP-1 and related agents on vascular endothelial function. These findings included: 1) MK-626 reversed the decreased lncRNA ENSMUST00000155383 in the aortae and ECs of hypertensive mice; 2) MK-626 restored Cacna1c mRNA and protein expression in the aortae and ECs from hypertensive mice; and 3) MK-626 resulted in the elevation of intracellular Ca^2+^ concentration in hypertensive mouse ECs. In order to further clarify the role of lncRNA ENSMUST00000155383, we manipulated siRNA delivery to the aortic ECs from the hypertensive mice with MK-626 remedy. Silence of lncRNA ENSMUST00000155383 reduced Cacna1c mRNA level and protein expression. Furthermore, the siRNA also caused the decrease of Ca^2+^ concentration and the suppression of eNOS phosphorylation in the ECs from Ang Ⅱ and MK-626 co-treatment mice. These data implied that the protective effect of lncRNA ENSMUS0000155383 on vascular endothelial cells may be related to the increase of intracellular Ca^2+^ concentration mediated by L-type Ca^2+^ channel and the subsequent activation of eNOS. In order to confirm the regulatory manner of GLP -1 and related agents on lncRNA ENSMUST00000155383 expression, we cultured the aortic ECs from C57BL/6J mice with GLP-1 receptor (GLP-1R) agonist exendin-4 and GLP-1R antagonist exendin 9–39 and then examined lncRNA ENSMUST00000155383 levels by qRT-PCR. As shown in Figure S6, exendin-4 increased the level of lncRNA ENSMUST00000155383 in the ECs, while exendin 9–39 inhibited the increase of lncRNA ENSMUST00000155383 induced by exendin-4, indicating that GLP-1/GLP1R activation could increase lncRNA ENSMUST00000155383 expression in a direct manner. Numerous studies suggest that GLP-1 and its analogues could modulate lncRNA expression profiles in many diseases including diabetes (Huang et al., 2020) and atherosclerosis (An et al., 2020). LncRNAs regulate gene expression at the transcriptional and post-transcriptional levels. Therefore, we’d better manipulate some new experiments to reveal the regulatory mechanism of lncRNA ENSMUST00000155383 on its downstream *Cacna1c*. However, this is beyond the scope of the present study.

## Conclusion

**FIGURE 8:**
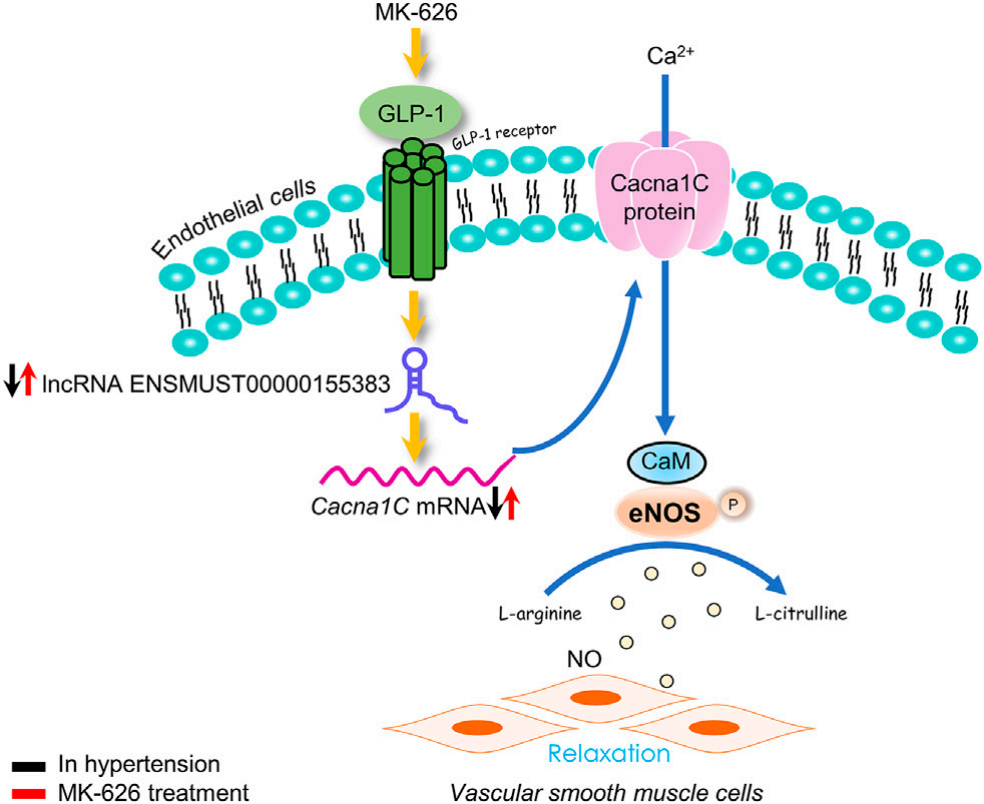
The proposed molecular mechanism for the beneficial effect of sitagliptin on endothelial function in hypertension. The eNOS activity was suppressed in hypertension. Elevation of GLP-1 by MK-626 stimulated eNOS activation via restoring cytosolic Ca^2+^ concentration through lncRNA ENSMUST00000155383/Cacna1c, leading to the improvement of endothelial function in hypertension.

This study highlights the dysregulations of lncRNA ENSMUST00000155383-associated genes in the vascular endothelium and demonstrated that MK-626 may improve vascular endothelial function in hypertension through upregulating lncRNA ENSMUST00000155383, restoring Ca^2+^ entry mediated through Cacna1c, subsequently enhancing eNOS activity (Figure 8). Thus, the present study provides new strategy for the pathogenesis and treatment of hypertension-related endothelial dysfunction.

## Data Availability

The datasets presented in this study can be found in online repositories. The names of the repository/repositories and accession number(s) can be found below: https://www.ncbi.nlm.nih.gov/geo/query/acc.cgi?acc=GSE165561.
