# Supplementary material for: LncRNA ENSMUST00000155383 is Involved in the Improvement of DPP-4 Inhibitor MK-626 on Vascular Endothelial Function by Modulating Cacna1c-Mediated Ca2+ Influx in Hypertensive Mice
Source: Front Mol Biosci. 2021 Jul 23;8:724225. doi: 10.3389/fmolb.2021.724225 (PMC8343177; doi:10.3389/fmolb.2021.724225)
Supplement: Supplementary file 1 [file DataSheet1.pdf]

Supplementary Material

Supplementary Figures

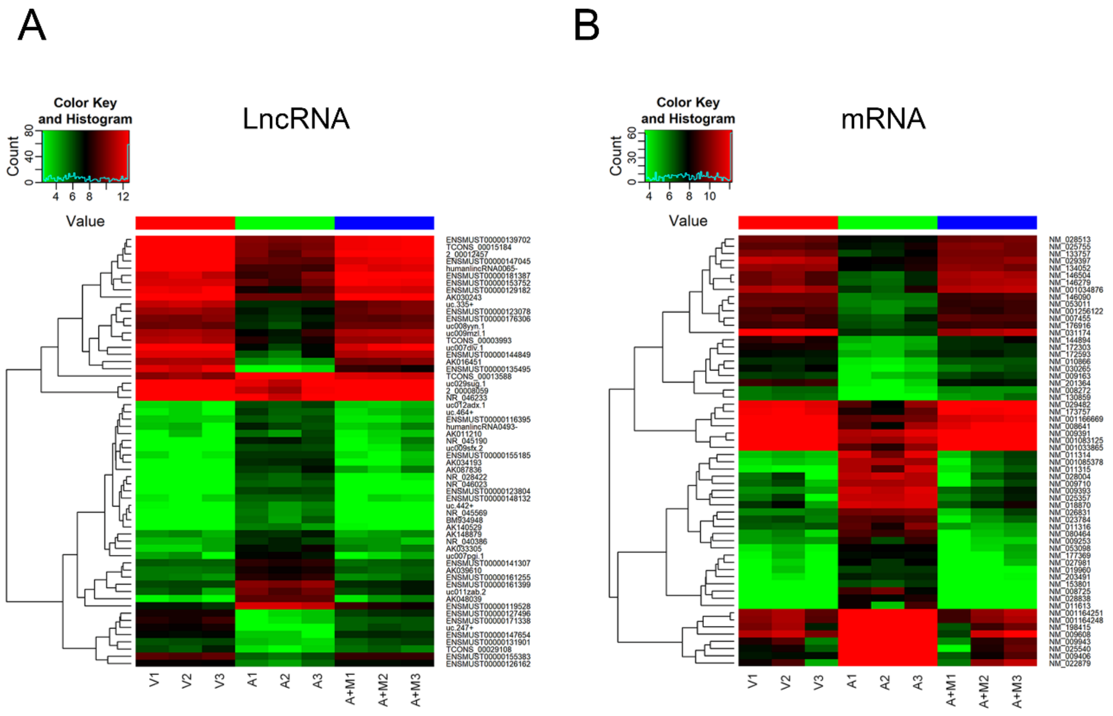

Supplementary Figure 1. Hierarchical clustering analysis of differentially expressed genes. Heatmaps for 60 DE-lncRNAs **(A)** and 60 DE-mRNAs **(B)** in the three groups. V, Vehicle; A, Ang II; A+M, Ang II+MK-626; DE-lncRNAs, differentially expressed lncRNAs; DE-mRNAs, differentially expressed mRNAs.

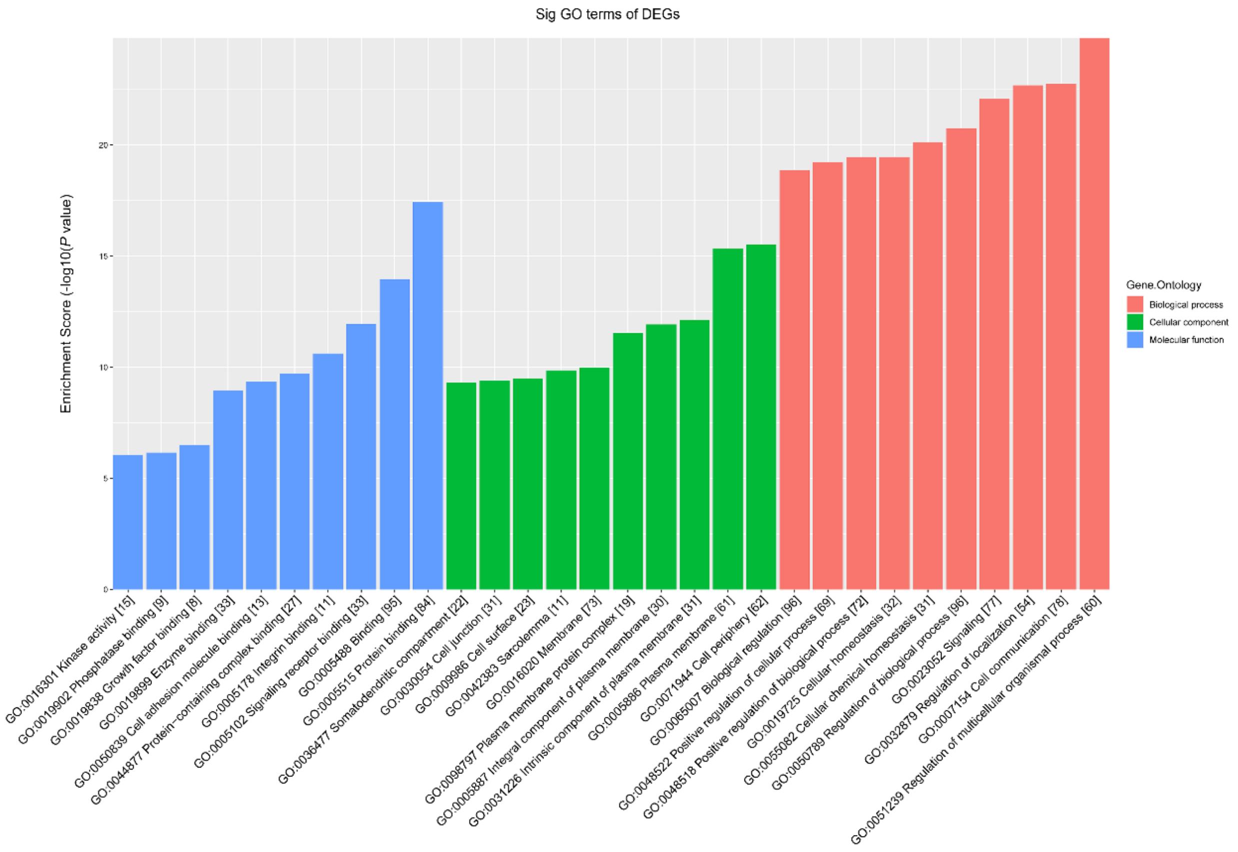

Supplementary Figure 2. The top 10 GO terms associated with differentially expressed genes (DEGs) in the coding-noncoding gene co-expression analysis. GO, gene ontology.

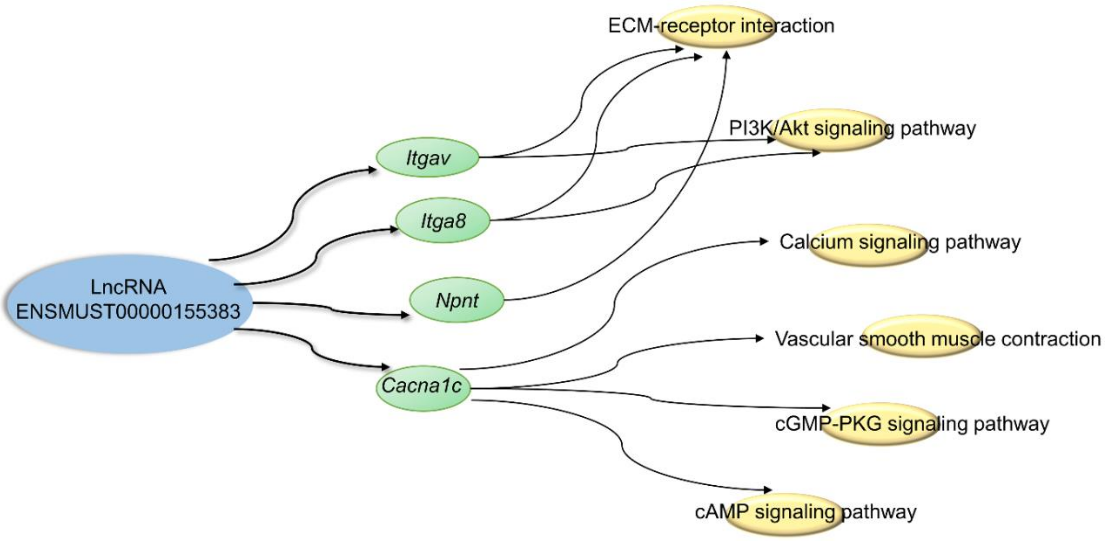

Supplementary Figure 3. Pathway analysis of lncRNA ENSMUST00000155383 and the candidate mRNAs. The network reveals that *Cacna1c* is involved in calcium signaling pathway, vascular smooth muscle contraction, cGMP-PKG signaling pathway, and cAMP signaling pathway. *Itgav*, *Itga8*, and *Npnt* participate in the ECM-receptor interaction. *Itgav*, and *Itga8* are related to the PI3K/Akt signaling pathway. The blue ellipse indicates lncRNA ENSMUST00000155383; the green ellipses represent downregulated genes induced by Ang II but reversed by MK-626; the yellow ellipses represent the related signaling pathways.

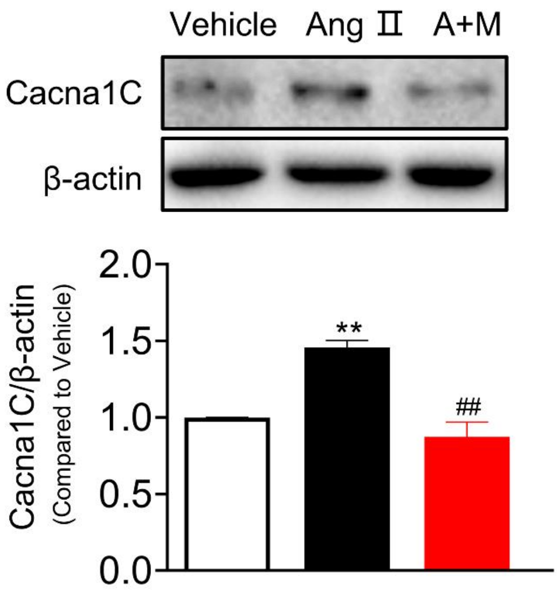

Supplementary Figure 4. The Cacna1c expressions in mouse aortic smooth muscle cells. Protein expression of Cacna1c was upregulated in mouse aortic smooth muscle cells from hypertensive mice, which was inhibited by MK-626. $^{**}P < 0.01$ *vs.* Vehicle; $^{\#\#}P < 0.01$ *vs.* Ang II. Data are expressed as mean ± SEM. n=3. A+M, Ang II+MK-626.

.

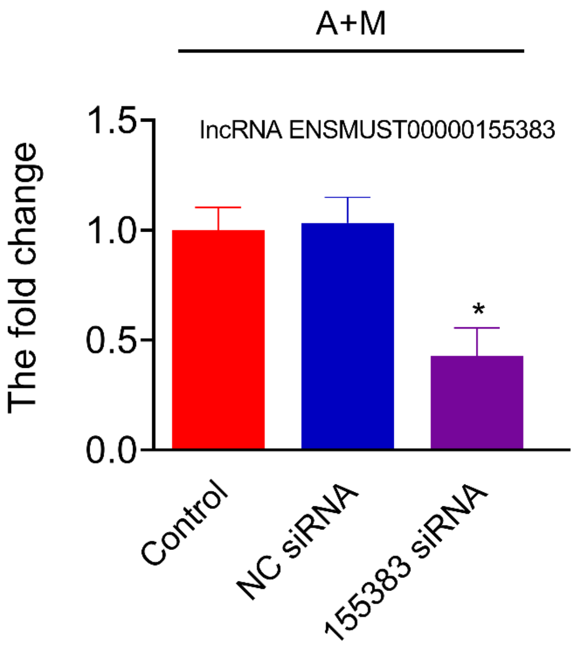

Supplementary Figure 5. The identification of siRNA knockdown efficiency. Using siRNA delivery, we achieve >60% reduction in the lncRNA ENSMUST00000155383 level in aortic ECs from hypertensive mice after MK-626 treatment. n= 4~5. A+M, Ang II+MK-626.

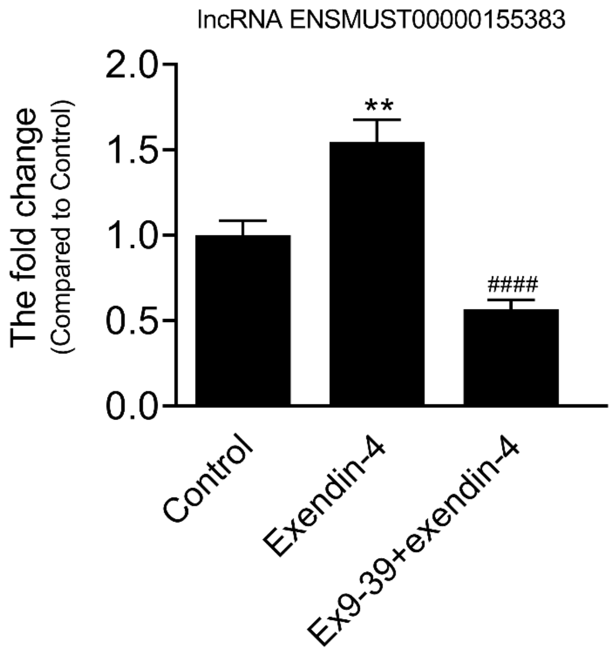

Supplementary Figure 6. Effects of exendin-4 with or without exendin 9-39 on the level of lncRNA ENSMUST00000155383 in the aortic ECs from C57BL/6J mice. $^{**}P < 0.01$ *vs.* Control; $^{\#\#\#\#}P < 0.0001$ *vs.* Exendin-4. Ex9-39, exendin 9-39. Data are expressed as mean ± SEM. n=7

Supplementary Table 1. The primer sequences of lncRNAs in the present study.

Gene name	Forward primers (5'-3')	Reverse primers (5'-3')
NR028422	AATGGTAGAGAGGCACCTGACTG	CAGATTCAGTTCTCTTCAAGGGTG
NR046023	TTAGTGCTGGAGATTAGTCCACTGC	GTAGTTGTGGCTGTGGATGTTCC
NR045569	CGTCAGAGCCATCCAGAATAACA	AGGAGTACTCTCTTTCCTTCTGGGA
ENSMUST00000116395	GAAACTATTTGCCTTGTTACCCATG	GTGGTATACCCACTGACGATCCTC
ENSMUST00000161399	GATGGTTCGGTGCATGAAGG	TCAGAAGCCAAGCAAGTCCTC
ENSMUST00000155185	TCTCTGGACAATGAAGTCTGCC	AGATATGCCCTGACTCTTGCAAA
ENSMUST00000161255	TCCATCAGTTAATGGGGAGTAGTAG	TTCCTCGATAGACAGCAAAATCC
uc007pgi.1	GATGAATGTGACCTGGGGAAAG	CAACTGGCTGCTCATGGTGTAC
uc009sfx.2	GGTAACGGCTCGTGCTAACTG	CCCGCAGGTGTACATTTATCTAG
uc009mzl.1	CCATCAAACTGGATTCACCTGT	ATCATTTTCTGGGAAAGAGCTG
AK030243	AATTGGCAACTGATTGGATGTC	TGTGAACTACTAAGGTCCCCACTTT
ENSMUST00000144849	AAGCAACCAAGAGTCTGGAAACC	ATCATCCCTGGCATCCTGAAC
ENSMUST00000147654	TCATTCACTTCCTCCTCTACATGCT	TTGCCTAATCCTCTGTAAAGATGCT
ENSMUST00000153752	GGACCCACCCATACTAGGCA	CTCGGAAAGTCTGGCGCTCT
ENSMUST00000155383	CTGCCTCAGTTTACCCACCAC	GCACAGCAACACCAACTACAGA
uc335+	AACCACTGGGACCTGAGACC	GGCCATGCATTACTGAACTTG
uc247+	ATAGCGGTATCGATTGATCCTG	AAGCACTGGGCAATAAAACTCA

Supplementary Table 2. The primer sequences of mRNAs in this study.

Gene name	Forward primers (5'-3')	Reverse primers (5'-3')
Tnni3	TCTGCCAACTACCGAGCCTAT	CTCTTCTGCCTCTCGTTCCAT
Chrm2	CGGACCACAAAAATGGCAGGCAT	CCATCACCACCAGGCATGTTGTTGT
Mylk3	AGAAGAGCTGTAGGATGAGCT	TTTCCGTAGCCTGTTGACTG
Il-6	TAGTCCTTCCTACCCCAATTTCC	TTGGTCCTTAGCCACTCCTTC
Cacna1C	TGATCCTGAAGCTCATTGCCT	TGCTCCCAATGACGATGAGG
Itgav	AAGGCGCAGAATCAAGGGGA	CCAGCCTTCATCGGGTTTCC
Itga8	ATCTCTTGTGCAGTGGGTCG	TTCTTTCTCTTGAGGAACGTGT
Npnt	GAAGCCTCGGCCCTGTAAG	AGCATGTATCCGTTGAGACAGTA
GAPDH	TTCCAGTATGACTCCACTCACG	CCTCACCCCATTTGATGTTAGT

Supplementary Table 3. The co-expression gene number of 13 DE-lncRNAs

Gene name	Co-expression gene number
ENSMUST00000155383	69
ENSMUST00000144849	60
AK030243	57
ENSMUST00000147654	54
NR_045569	52
ENSMUST00000161399	50
ENSMUST00000155185	41
ENSMUST00000161255	39
NR_046023	39
NR_028422	35
uc009mzl.1	39
ENSMUST00000116395	26
ENSMUST00000153752	10

Supplementary Table 4. The normalized microarray value of candidate mRNAs.

Name	Seqname	Chrom	V1	V2	V3	A1	A2	A3	A+M1	A+M2	A+M3
Itgav	NM_008402	chr2	7.368589	7.219565	7.541989	5.626201	5.599203	5.864886	7.393943	7.20308	7.195142
Itga8	NM_001001309	chr2	10.95263	10.84623	11.08958	8.735723	8.291734	9.111872	10.81214	10.62364	10.28179
Npnt	NM_033525	chr3	8.628272	8.382823	8.868811	6.595824	6.385525	6.959857	8.753069	8.257404	8.056559
Cacna1c	NM_009781	chr6	4.470658	4.559325	4.496615	2.354583	2.327623	2.857156	4.154073	4.420234	4.010129
Tnni3	NM_009406	chr7	7.552876	7.69621	7.672329	12.57489	12.5574	12.90284	6.345742	8.109036	9.22786
Mylk3	NM_175441	chr8	5.323415	5.319858	5.520079	8.565525	8.808997	7.691926	4.063713	5.079677	5.628933
Chrm2	NM_203491	chr6	2.529607	3.950833	3.409739	7.406296	7.079491	7.274245	2.330085	3.394942	3.704214
Il6	NM_031168	chr5	4.770724	5.245423	5.069062	7.852998	6.155218	8.494091	4.827996	4.594006	4.581033

V, Vehicle; A, Ang II; A+M, Ang II+MK-626.
